# Implementation of interprofessional quality circles on deprescribing in Swiss nursing homes: an observational study

**DOI:** 10.1186/s12877-023-04335-w

**Published:** 2023-10-03

**Authors:** Stephanie Mena, Joanna C. Moullin, Marie Schneider, Anne Niquille

**Affiliations:** 1 Community Pharmacy, Center for Primary Care and Public Health (Unisanté), University of Lausanne, Rue du Bugnon 44, CH-1011 Lausanne, Switzerland; 2 School of Pharmaceutical Sciences, University of Geneva, Geneva, Switzerland; 3 Institute of Pharmaceutical Sciences of Western Switzerland, University of Geneva, University of Lausanne, Geneva, Switzerland; 4 Faculty of Health Sciences, Curtin University, Curtin School of Population Health, Perth, Australia

**Keywords:** Implementation science, Implementation outcomes, Implementation strategies, Quality circle, Deprescribing, Nursing homes, Potentially inappropriate medication

## Abstract

**Background:**

Polypharmacy and potentially inappropriate medications (PIMs) are still frequent among older adults in nursing homes. Deprescribing is an intervention that has been shown to be effective in reducing their use. However, the implementation of deprescribing in clinical practice has not yet been widely evaluated. The Quality Circle Deprescribing Module (QC-DeMo) intervention has been trialled through an effectiveness-implementation hybrid type 2 design. The intervention consists of a quality circle workshop session between healthcare professionals HCPs (physicians, nurses, and pharmacists) within a nursing home, in which they define a consensus to deprescribe specific PIMs classes. The aim of this study was to evaluate the implementation of the QC-DeMo intervention in nursing homes.

**Methods:**

This observational study focuses on the implementation part of the QC-DeMo trial. Implementation was based on the Framework for Implementation of Pharmacy Services (FISpH). Questionnaires at baseline and follow-up were used to evaluate reach, adoption, implementation effectiveness, fidelity, implementation, maintenance and the implementation strategies. Other data were collected from the QC-DeMo trial and routine data collected as part of the integrated pharmacy service where the QC-Demo trial was embedded. Implementation strategies included training of pharmacists, integration of the intervention into an existing quality circle dynamic and definition of tailored strategies to operationalise the consensus by each nursing home.

**Results:**

The QC-DeMo intervention was successfully implemented in 26 nursing homes in terms of reach, fidelity, adoption, implementation and implementation effectiveness. However, the intervention was found to be implemented with low maintenance as none of the nursing homes repeated the intervention after the trial. Implementation strategies were well received by HCPs: training was adequate according to pharmacists. Pre-existing quality circle dynamic facilitated interprofessional collaboration as involvement and support of each HCP was rated as high. HCPs recognized a specific and important role for each HCP in the deprescribing process. The most relevant tailored strategies to implement the consensus defined by each nursing home were identification of the patients by the pharmacist and a systematic review of medication’s patients.

**Conclusions:**

The implementation of a Quality Circle on Deprescribing is feasible but its maintenance in practice remains challenging. This study explores multiple implementation outcomes to better inform future implementation efforts of these types of interventions.

**Trial registration:**

ClinicalTrials.gov (NCT03688542), registered on 26.09.2018.

**Supplementary Information:**

The online version contains supplementary material available at 10.1186/s12877-023-04335-w.

## Background

Polypharmacy, generally defined as the use of five or more daily medications [1], along with the use of potentially inappropriate medications (PIMs) are common among the aging population, especially in nursing homes [2]. Both are a concern due to their association with adverse effects such as falls or cognitive impairment [3–6]. Over the past decade, deprescribing, the process of withdrawal or dose reduction of an inappropriate medication supervised by a health care professional (HCP) [7], has become a safe intervention to deprescribe medications of older adults [8, 9]. Multiple large-scale interventions have been shown to be effective in reducing PIMs use in this population [10–14].

In Switzerland, there were 1542 nursing homes in 2020 including 132 in the canton of Vaud and 40 in the canton of Fribourg [15]. The role of the various HCPs in nursing homes differs. The physicians who are responsible for patients in nursing homes are mostly general practitioners, who continue to follow their patients once they have arrived in the nursing home or who act as referring physicians for the majority or entire nursing home. The referring pharmacist is responsible for delivering medications to patients throughout the nursing home, by agreement with the director of the nursing home. Other activities attributed to the referring pharmacists include the delivery of monitored dosage systems or the management of narcotics. Finally, nurses, unlike other HCPs, are directly employed by the nursing home and provide daily medical care at the patients’ bed [16]. Since 2009 in nursing homes of the canton of Vaud and between 2002 and 2018 in nursing homes of the canton of Fribourg [17], an integrative pharmacy service (IPS) was established to promote the rational use of medication through quality circles [18]. Quality circles consist of meetings in which the three different HCPs, i.e. nurse, pharmacist and physician, in the nursing home discuss how to improve their practice and develop local guidelines, based on evidence-based medicine and annual drug data use within the nursing home. The quality circles are led by the pharmacist and a consensus on the clinical guidelines to be followed is defined by the HCPs of the nursing home. Its implementation is evaluated the following year based on the updated medication consumption data.

Since this service is funded by the cantons ‘ health department, participating nursing homes are required to hold at least one annual session and to submit an annual report on their activities and annual coded drug data. This report includes a description of the consensus and an evaluation of their achievements in the past year. The IPS project is being monitored by a Unisanté pharmacy research team.

The Quality Circle Deprescribing Module (QC-DeMo) was developed in Switzerland. It is embedded into the IPS and uses the same quality circle methodology. QC-DeMo’s effectiveness has been reported elsewhere [19]. The intervention did not reduce the primary outcome, i.e. the total number of PIMs per resident, measured using both the “Avoid” and “Reevaluate” categories of the validated Beers [20] and NORGEP-NH [21] tools. Nevertheless, a reduction was observed in the number of doses of PIMs per resident, measured in Defined Daily Dose [22].

Deprescribing interventions, such as QC-DeMo, are complex interventions, as they contain several interacting components to be deployed in multiple locations, requiring a multi-level implementation strategy [23]. Evaluation of implementation processes and outcomes are seen as an important component of improving the uptake of evidence-based interventions in routine clinical practice [24–26]. Many studies have been conducted to evaluate the implementation of interventions to optimise the use of medicines in nursing homes [27–31]. Most of those studies evaluated some aspect of the implementation (e.g., barriers and facilitators or implementation strategies) through a qualitative study, process evaluation or quality improvement study [32, 33]. However, the simultaneous evaluation of multiple implementation outcomes, such as adoption, fidelity, reach, or maintenance in nursing homes, such as analyzed in this present paper, has not yet been evaluated in this context. Therefore, using an implementation science approach to explore the unique barriers in a nursing home setting to inform future implementation efforts to reduce PIMs is needed.

The aim of this study was to evaluate the implementation of the QC-DeMo intervention in nursing homes.

## Method

### Design

This study is part of an effectiveness-implementation hybrid type 2 design trial [34]. The effectiveness evaluation was conducted through a randomized controlled trial (RCT) reported elsewhere [19]. This part focuses on the evaluation of the implementation of the QC-DeMo intervention through an observational study.

### QC-DeMo intervention

**Fig. 1:**
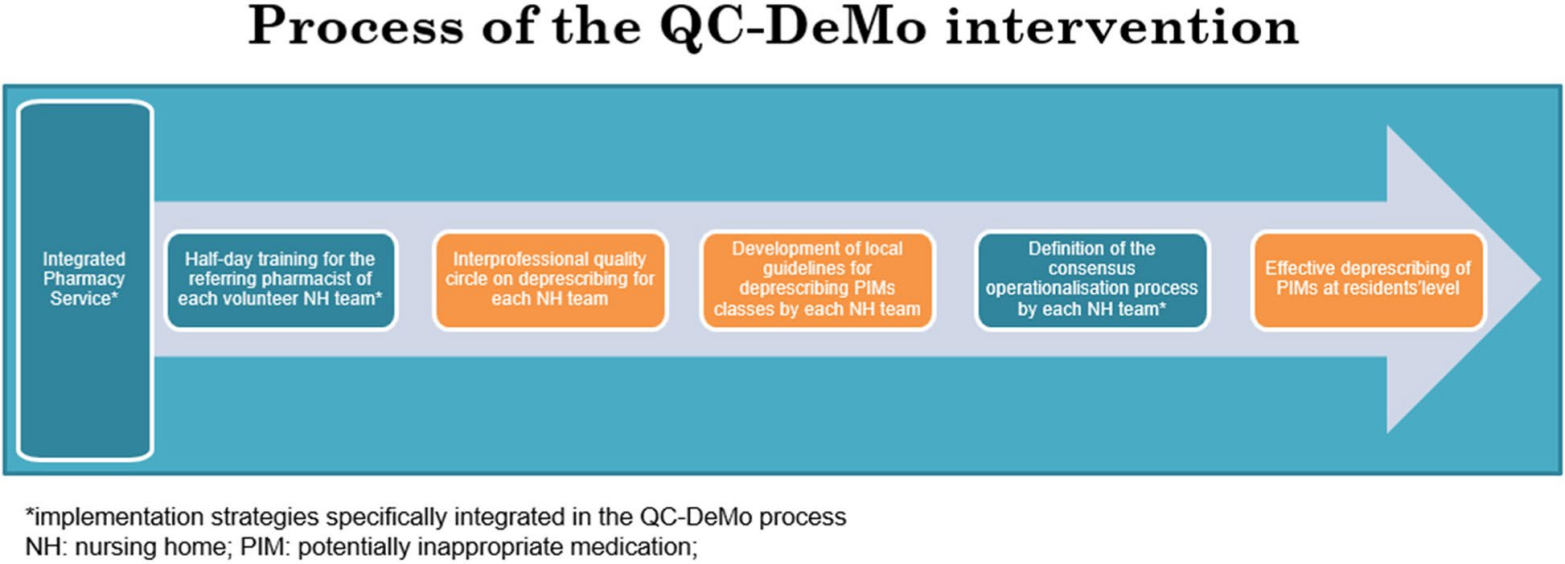
Process of the QC-DeMo intervention

QC-DeMo consists of an interprofessional quality circle session between a physician, pharmacist, and nurse of each participating nursing home team. The core content of the intervention is a pharmacist-led session to develop guidelines within the nursing home for deprescribing potentially inappropriate medications (PIMs) classes. This is operationalised by a structured evaluation of PIMs consumption by the nursing home residents, based on pharmacy records. Local PIMs data are compared to safety evidence from literature. At the end of the session, HCPs are asked to reach a consensus and to prioritise which PIMs drug classes to deprescribe, based on the prevalence of use within their nursing home. A detailed figure of the intervention and implementation strategies is shown in Fig. 1.

### Setting and participants

The QC-DeMo intervention was offered to all nursing homes in two cantons (Vaud and Fribourg) of the French-speaking part of Switzerland, who had been active in the integrated pharmacy service for at least one year at the time of the recruitment. The intervention was conducted over a year, in two rounds: the first round where nursing homes performed a QC-DeMo session took place in December 2017 and the second one in December 2018. For each round, the impact on PIM use was measured between the year before the QC-Demo session (baseline) and the year after, as part of the effectiveness study, where a more detailed description of the QC-DeMo intervention is reported [19, 35].

### Implementation outcomes

The implementation process and evaluation were built on the Framework for the Implementation of Services in Pharmacy (FISpH) [36] and Reach, Effectiveness, Adoption, Implementation, Maintenance framework (RE-AIM) [37] using Proctor’s et al. taxonomy [38]. The FISpH, is an implementation science framework designed specifically for pharmacy services. It consists of implementation stages, contextual levels and tailored Consolidated Framework for Implementation Research [39] determinants for pharmacy settings. It was used to plan and design the implementation study, while the RE-AIM was used to structure the evaluation. Each outcome was categorized according to the stage of implementation: RE-AIM is a multilevel framework often used to evaluate the implementation of interventions, whereby the acronym stands for the measures Reach, Effectiveness, Adoption, Implementation, and Maintenance. However, the proposed chronological sequence for using these measures is ARIEM or adoption, reach, implementation, effectiveness and maintenance. In our study we adapted the term of the "effectiveness" dimension to "effectiveness of implementation" to avoid confusion with the RCT effectiveness reported elsewhere. Adoption is defined as the number and proportion of eligible participants (healthcare professionals) who initiated the intervention. Reach is defined as the number and proportion of eligible population (nursing home residents) who received the intervention. Implementation effectiveness is defined as the impact of the intervention on individual outcomes and variability between subgroups. Implementation is defined as the participants’ use of the intervention. Maintenance is defined as part of the extent to which an intervention becomes institutionalized or part of the routine organizational practices [37].

### Implementation strategies [40]

Three implementation strategies, categorized according to Powell et al. [41] and Perry et al. taxonomies [42] were: 1) pharmacist training, 2) integration of the intervention into an existing interprofessional quality circle and 3) definition of the consensus operationalisation process by each nursing home team.

The first implementation strategy, during the preparation phase, was a half-day pharmacist training workshop. The material covered focused on existing tools to detect PIMs including Beers [20], PRISCUS [43], STOPP-START [44] or NORGEP-NH [21] criteria and an overview of existing evidence on safety and effectiveness of ten PIMs drug classes, i.e. proton pump inhibitors (PPIs), statins, benzodiazepines & z-drugs, hypoglycaemics, urinary tract spasmolytics, neuroleptics, antidepressants, antidementia drugs, antihypertensives and bisphosphonates. A presentation template using Powerpoint software was also provided to the pharmacists to assist them in conducting the QC-DeMo session.

The second implementation strategy was to integrate the intervention into the existing IPS. Eligible nursing homes had to have been part of the IPS for at least one year at the time of the recruitment, thus interprofessional collaboration was already established before starting QC-DeMo. This interprofessional collaboration was a facilitator of implementation.

The third implementation strategy was to actively involve the nursing home’s quality circle teams in the development of the intervention by defining their own tailored strategy to operationalise the process. Indeed, each of them had to define a tailored strategy to facilitate the implementation of their consensus. Concretely, they had to define an approach for their own nursing home to effectively withdraw each class of selected PIMs. This involved defining and documenting each stage of the process, when it would be implemented, how and by whom.

### Outcomes measures

The implementation outcomes assessed by stage, their measures, and their data sources are described in Table 1.

**Table 1:** Implementation outcomes measures and data sources, by stage of implementation

Stages of implementation	Outcome	Measure	Data source
*Preparation*	Adoption - NH level	• Number and proportion of NH teams who volunteered to participate to the trial • Number of NHs who delivered a QC-Demo session • Percentage of nursing homes already performing activities related to deprescribing prior to the intervention • Percentage of NH team reporting that deprescribing measures were already implemented prior to the intervention	QC-DeMo monitoring Questionnaire baseline (T0)
	Adoption- HCP level	• Number of participating HCPs in the intervention • Number of years of collaboration within the NH for referring physicians and pharmacists, and employed nurses • Previous training on deprescribing	Attendance list Questionnaire baseline (T0)
*Operation*	Reach	• Number of residents who received a PIM from the selected PIM classes chosen by all NHs	IPS and QC-DeMo monitoring
	Implementation effectiveness	• Number of NHs that reduced their average DDD per resident between the year after the intervention and to the year before, compared to the control group • Number of residents who reduced their PIM use in DDD during the year after the intervention, compared to the control group • For each PIM class selected: percentage of residents who had reduced their PIM use in DDD	IPS monitoring
	Implementation	• HCP’s perception of the QC-DeMo session (quality, comprehensiveness, relevance, consensus) • Level of importance to deprescribe as perceived by HCPs • Barriers and facilitators to reaching and implementing deprescribing consensus • HCPs' global satisfaction with the intervention • Proportion of HCPs who would recommend other NHs to adopt a similar process	Questionnaire baseline (T0) and follow-up (T12)
	Fidelity	• Number of NHs that achieved at least one consensus and at least one implementation strategy established following the session • Number of NHs that selected 50% or more of their five most prevalent PIMs drug classes for deprescribing • Comparison of the use in DDD per average resident of each PIMs classes between NHs that included that PIM class in their consensus and those who did not at baseline	QC-DeMo monitoring IPS monitoring and QC-DeMo monitoring
*Sustainability*	Maintenance	• Proportion of physicians who believed they have sustainably changed some of their prescribing practices through the intervention • Proportion of physicians and nurses who would find a further QC-DeMo session useful • Proportion of pharmacists who intended to plan a second QC-DeMo session • Number of NHs that renewed a QC-DeMo session and/or had rediscussed the defined consensus • Number of participating NHs who reduced their overall PIMs two years after the intervention	Questionnaire (T12) IPS monitoring

*DDD* Defined Daily Dose as defined by the World Health Organization as the ATC/DDD Index, *HCP* healthcare professional, *IPS* integrated pharmacy service, *NH* nursing home, *PIM* potentially inappropriate medication, *QC-DeMo* Quality Circle Deprescribing Module

### Data collection

Two different questionnaires were designed and administered at two points: one questionnaire at the end of the QC-DeMo session (baseline, T0) and the other, one year after (follow-up, T12). Specific version of the questionnaires was designed for each health care professional (i.e., nurses, pharmacists and physicians). The baseline was based on three themes 1) previous deprescribing experiences of HCPs, 2) perception of implementing deprescribing measures within the nursing home and 3) perception and satisfaction of HCPs concerning the QC-DeMo they attended for nurses and physicians and led for pharmacists. A fourth section evaluated the two first implementation strategies by asking questions on pharmacists’: perception regarding the training they received before delivering the intervention and interprofessional collaboration, by asking the importance of each other ‘s role in the deprescribing process before the implementation of the consensus.

To assess the implementation process and evaluation, the follow-up questionnaire was built on three themes: 1) evaluation of the process (satisfaction, barriers, and facilitators to drafting the deprescribing consensus), 2) maintenance of the intervention and 3) barriers and facilitators to the implementation of the consensus. A fourth section evaluated the second and third implementation strategies: interprofessional collaboration by asking perceived degree of involvement and support of each HCPs and the perceived respective role. The second strategy evaluated in this section was the description of the operationalisation process defined by each team and perceived effectiveness of their process.

To ensure that the content of the questionnaires was adapted to the real-world setting, their face validity was conducted by one experienced HCP who regularly worked with the research group. The baseline questionnaires were printed by the investigators and given to the pharmacists at the beginning of the trial in the study binder containing all the intervention documentation. The follow-up (T12) questionnaire was sent by mail. The detail of each theme, items and type of scale of responses used is available in Additional file 1.

An information sheet about the process and a corresponding consent form were included with the baseline questionnaire provided to all HCPs. An attendance list was given to each nursing home for all HCPs who participated in the QC-DeMo session to sign. The absence of physicians and the reason for it were also noted on this list. To ensure confidentiality, pre-stamped envelopes with a nursing home code system were used to collect questionnaires at baseline and follow-up. Results from questionnaires consider the two rounds of QC-demo sessions, i.e., the ones which took place during the year 2017 and the ones during the year 2018.

Additional data on implementation outcomes were obtained from the monitoring of the QC-DeMo trial and the monitoring of the IPS. Consensus with selected PIM classes and the attendance list were collected through the monitoring of the QC-DeMo trial. Drug use data and annual activity reports from each nursing home at baseline and up to two years after the intervention were collected through the IPS monitoring.

In 2018, the IPS in the Canton of Fribourg was announced ended, and as such only data from the IPS of the Canton of Vaud were available to measure the number of participating nursing homes (*n* = 21) who reduced their overall PIM, using Beers and NORGEP-NH criteria, 2 years after the intervention (maintenance).

Table 2 presents the category of implementation strategies, their description, their measures, and their data source.

**Table 2:** Implementation strategy descriptions and measures

Implementation strategy	Description	Category	Measure	Data source	Implementation outcomes potentially affected
Foster the learning collaborative dynamic of the quality circle	Interprofessional collaboration to optimize the use of medicines	Educate through peers	• Perception of the importance of each respective professional role in the deprescribing process • Perception of the importance of interprofessionality in this approach • Degree of involvement and support of each HCPs role • Description of the role of each HCP	• T0 • T0 • T12 • T12	• Implementation • Maintenance
Conduct educational meetings for pharmacists and distribute educational materials for pharmacists	Training for pharmacists	Educate strategy	• Training evaluation by pharmacists • Utility of the template presentation	• T0 • T0	• Adoption • Fidelity • Maintenance
Prepare HCPs to be active participants of the change	Definition of tailored strategy to operationalise the process by each NH team	Engage HCPs	• Description of the tailored strategies after a year • Effectiveness of the tailored strategies reported by HCPs	• T12 • T12	• Fidelity • Maintenance

*HCP* healthcare professional, *NH* nursing home, *T0* questionnaire at baseline, *T12* questionnaire at follow-up

### Data analysis

All data collected by paper questionnaires were entered by a research assistant pharmacist into an excel database and double-checked by the main researcher (SM).

#### Implementation outcomes

Descriptive statistics (number, proportion, percentages, means and standard deviations, medians, and interquartile range) were calculated for adoption, fidelity, reach, implementation, and maintenance. Fidelity was defined as follows: as the nursing home teams had to choose the highest prevalence of PIMs within their nursing home, fidelity was measured as the number of nursing homes that chose these classes. We then compared if there was a difference in PIMs at baseline between the nursing homes that chose the most prevalent PIMs and those who did not choose those PIMs classes. For measuring fidelity, the PIMs classes selected for deprescribing were compared with data consumption of the nursing home collected through IPS monitoring. The five most prevalent PIMs classes in each nursing home were identified. Fidelity was considered as high if 80% or more [45, 46] of the selected classes by the nursing home during the QC-DeMo session were among the most prevalent PIMs classes. To compare in the use of PIMs classes at baseline between the nursing homes that selected them and those who did not, we separated the nursing homes in two corresponding groups. Unpaired sample t-tests were then performed for each PIM drug class discussed in the QC-DeMo sessions to test the difference in PIMs use at baseline between the two groups. A *p*-value at < 0.05 was considered as statistically significant. Statistical analyses were conducted in Stata, version 16. To measure the outcome called implementation (see Table 1), the quality of the QC-Demo session was scored by nurses and physicians, on a scale of 0 to 10, where 0 = lowest quality and 10 = highest quality.

DDD per resident were measured using the same methodology as described in the effectiveness study [19]. PIMs drug classes were classified in two categories Avoid and Reevaluate, using the validated Beers [20] and NORGEP-NH [21] tools. The two categories Avoid and Reevaluate were used to measure the average Defined Daily Dose per resident. The formula is presented as followed:

$$\frac{\left(number\;of\;boxes\;delivered\right)\;x\;\left(number\;of\;units\;per\;box\right)\;x\;(dose\;per\;unit)}{Defined\;Daily\;Dose\;x\;(number\;of\;days\;spent\;in\;the\;NH\;during\;the\;year)}$$

HCPs global satisfaction and their perception of the importance to deprescribe in nursing home were measured on a scale of 0 to 10, where 0 = lowest level of importance and 10 = highest level of importance.

The barriers and facilitators to drafting the deprescribing consensus were regrouped from each HCPs questionnaire. They were merged into themes if they occurred more than once. Those that appeared only once but were considered particularly relevant by the investigators were also reported. Barriers and facilitators to the implementation of the deprescribing consensus were also regrouped from each HCP questionnaire and merged into themes. A barrier or a facilitator to a particular PIM drug class was considered as relevant by investigators, it was also reported.

#### Implementation strategies

To analyse the degree to which interprofessional collaboration facilitated implementation, we measured the degree of involvement/support of each HCP on a scale of 0 to 10, where 0 = lowest level of involvement/support and 10 = highest level of involvement/support. We also measured the perceived importance of each HCP’s role, whereby each HCP was asked to score each HCP’s role from major to minor in the deprescribing process on a scale of 1–4, where 1 = major, 2 = important, 3 = minor and 4 = no opinion.

Nurses' and physicians' perceptions of the importance of interprofessionality in this approach were measured by rating interprofessionality as unnecessary, useful, or essential. Descriptive statistics were used to report the scores and illustrated with some qualitative insights from the free text collected through the questionnaires.

To evaluate pharmacists’ training, each pharmacist was asked to rate from 0 to 10 the quality of the training they received to deliver the intervention, where 0 = lowest quality and 10 = highest quality. To measure the utility of the template presentation, pharmacists were asked to rate the extent to which they had to modify the template to present the QC-DeMo session.

To identify the tailored strategies perceived as most relevant, HCPs were asked to rate the perceived effectiveness of each tailored strategy in the questionnaires, using a score of 0 to 10, where 0 = is the lowest perceived effectiveness of the strategy and 10 is the highest. The ones with a score of 10 were then regrouped into themes and resumed by each step of the deprescribing process.

## Results

### Implementation outcomes

Implementation outcomes are presented in Table 1.

### Adoption

#### Nursing home level

Of the 115 eligible nursing homes, 40 (35%) volunteered for the study during the first round, and 16 of 119 eligible nursing homes (13%) during the second round, which corresponds to 56 out of 119 potential nursing homes. New nursing homes became eligible (at least active in the IPS for one year) during the second round, which explains why the number of eligible nursing home rose from 115 to 119 between the two rounds. Across the two rounds, 27 nursing homes were assigned to the intervention group and 29 to the control group. In the intervention group, one nursing home did not deliver a QC-DeMo session, due to a health issue of the pharmacist.

Among the remaining 26 participating nursing homes in the intervention group, 48% (*n* = 12/25) reported having conducted, in addition to the quality circle session, simple medications reviews, 36% (*n* = 9/25) advanced medication reviews [47], and 44% (*n* = 12/25) reported that they conducted one or more additional quality circle sessions per year in addition to the required one.

Most HCPs (90% of physicians (37/41), 80% of pharmacists (20/25) and 75% of nurses and allied health professionals (38/51), reported that they had already implemented deprescribing measures in their nursing homes in the past.

#### HCP level

In total, 43 physicians, 45 nurses and 16 allied health professionals (community health or support assistants, coordinators, directors of care or of nursing homes) and 29 pharmacists attended a QC-DeMo session. All 26 nursing homes had at least one physician present during the QC-DeMo session, with an average of two physicians per session. Across all 26 nursing homes in the intervention group, a total of six physicians were absent (four unavailable and two not interested).

The mean duration of collaboration between HCPs within the nursing home was 15.0 years (SD: 12.5, range: 0–41) years for physicians, 8.7 years (SD: 8.8, range: 0–34) for nurses and 7.1 years (SD: 5.4, range: 0.5–26) for pharmacists.

Most HCPs (79% for nurses, 73% for pharmacists and 64% for physicians) had not participated in deprescribing training prior to participating in the intervention.

### Reach

The potential reach of the QC-DeMo intervention was 824 residents who across the 26 nursing homes in the intervention group, were being prescribed a PIM among the PIM classes selected by nursing homes.

### Implementation effectiveness

Nineteen of 26 nursing homes reduced their PIMs in terms of DDD per average resident one year after the intervention compared to 16 of the enrolled 28 nursing homes in the control group.

Compared with baseline, 294 residents reduced their total annual DDD of PIMs classes selected by nursing homes after the intervention among the 824 residents who received at least a PIM among the PIM classes selected by nursing homes. For proton pump inhibitors, residents reduced their PIM use in 27% of cases (105/394), in 25% (28/112) of residents taking statins, 27% (11/41) for urinary spasmolytics, 31% (71/228) for benzodiazepines and z-drugs, 32% (85/267) for antihypertensives, 33% (18/54) for hypoglycaemics, 44% (4/9) for bisphosphonates, 14% (1/7) for antidementia drugs, 18% (21/115) for antidepressants and 24% (11/46) for neuroleptics.

### Implementation

At baseline, the level of importance to deprescribe in their nursing home was rated as 7.9/10 by nurses (*n* = 54), 7.5/10 by physicians (*n* = 42) and 6.5/10 by pharmacists (*n* = 25). Most nurses and physicians rated the quality of the QC-DeMo session delivered by the pharmacists as good, with a mean score (± SD) of 8.0 ± 1.9 (*n* = 49) and 7.9 ± 1.7 (*n* = 37) respectively. Nurses and physicians also found that the quantity of information presented sufficient (48/51 and 36/40 respectively) and for the most part useful (27/52 and 20/38 respectively). Most physicians also reported that after the session, they intended to implement the entire consensus (30/38). In most cases, nursing home teams found that they easily reached consensus, 37/52 for nurses 22/38 for physicians and 10/21 for pharmacists.

At follow-up, the responses rate was 45% (13/29) for pharmacists, 28% (12/43) for physicians and 26% (16/61) for nurses. Overall satisfaction with the intervention was good in the view of responding HCPs with a mean score of 8.0/10 for physicians (*n* = 12) and both 7.4/10 for pharmacists (*n* = 12) and nurses (*n* = 16). And 14/15 nurses, 11/12 physicians and 9/13 pharmacists would recommend the intervention to other nursing homes. Barriers and facilitators reported by the HCPs to achieving a deprescribing consensus and to implementing the deprescribing consensus are presented in the Table 3.

**Table 3:** Barriers and facilitators to achieve and implement the deprescribing consensus

Achievement of the deprescribing consensus	
Barriers	Facilitators
**•** physicians’ resistance **•** lack of remuneration for physicians **•** pharmacist’s lack of knowledge of the resident **•** lack of time of all HCPs	**•** reflections on medications and their re-evaluation **•** exchange between HCPs: sharing knowledge and practices **•** structured process **•** training and material provided **•** reflections on healthcare costs
Implementation of the deprescribing consensus	
Barriers	Facilitators
**•** patient/family refusal **•** lack of diligence of physicians and/or availability **•** long-standing treatment difficult to modify • cognitive impairment of the residents	**•** motivation and involvement of nurses **•** consensus already in place for some drug classes (PPIs, antihypertensives, minerals and vitamins) **•** easy follow-up measures when deprescribing antihypertensives (e.g., blood pressure), collaborative work and shared vision by physicians and nurses **•** setting clinical glycemic targets when discontinuing hypoglycaemic**s**

### Fidelity

Following QC-DeMo session, all 26 nursing homes who received the intervention were successful in reaching at least one consensus of a PIM to deprescribe and in defining at least one operationalisation process to help the deprescribing of a drug class.

PIM prevalence in DDD per average resident did not differ between the nursing homes who chose the drug class as one to deprescribe and those who did not, except for spasmolytics and antidementia drugs. The difference (*p*-value) for spasmolytics was 0.038 (0.005), for antidementia drugs 0.042 (0.040), for proton pump inhibitors 0.029 (0.678), for statins 0.034 (0.116), for benzodiazepines 0.022 (0.403) and for z-drugs, 0.270 (0.054) for antihypertensives, 0.0163 (0.175) for hypoglycaemics, 0.005 (0.295) for bisphosphonates, 0.124 (0.977) for antidepressants and 0.030 (0.356) for neuroleptics.

The five most prevalent PIM classes in each nursing homes at baseline were identified. Only seven of the 26 nursing homes in the intervention group had between 80 and 100% of the PIM classes selected in the top 5 and six nursing homes had between 50 and 79% of the PIM classes selected in this list, as shown in Table 4 below.

**Table 4:** PIM classes (ATC codes) selected by each nursing home

NH Code	PPIs	Statins	Urinary Spasmolytics	BZDRAs	Anti-HT	Hypoglycaemics	Biphosphonates	Anti dementia	Anti depressants	Neuroleptics	No PIM class	top 5	DDD PIM/ average resident
	ATC codes												
	A02BC	C10	G04BD	N05B, N05C	C03, C07-09	A10B	M05BA, M05BB	N06D	N06A	N05A			
27	1	1	1	1			1	1			6	17	3.1
40	1	1		1	1					1	5	60	3.1
1	1			1	1						3	100	2.9
24	1			1					1		3	100	2.8
46		1			1	1					3	67	2.8
10	1	1		1	1					1	5	80	2.7
53		1				1		1			3	33	2.6
51	1		1	1							3	33	2.5
28	1	1	1								3	67	2.5
29		1	1		1		1				4	50	2.5
3	1	1	1								3	67	2.4
45		1	1						1	1	4	50	2.4
35	1	1	1					1			4	25	2.4
23	1			1					1	1	4	75	2.3
36	1	1	1			1		1			5	40	2.3
52		1				1		1			3	33	2.3
22	1			1					1	1	4	100	2.3
20	1					1	1				3	33	2.0
19	1					1	1				3	33	1.8
50	1	1		1				1			4	75	1.8
48	1	1			1						3	100	1.7
26	1	1		1	1	1			1		6	83	1.7
49	1	1	1				1				4	25	1.6
34	1	1			1	1					4	50	1.6
39	1								1		2	100	1.6
17	1					1	1				3	33	1.3
Total	21	17	9	10	8	9	6	6	6	5	Average number of PIM classes selected (mean (SD)): 3.7 (1.0)		

*AntiHT* antihypertensives, *ATC* Anatomical Therapeutical Chemical, *BZDRAs* Benzodiazepine Receptor Agonists, *DDD* Defined Daily Dose, *FR* Fribourg, *NH* nursing home, *PPIs* proton pump inhibitors, *PIM* potentially inappropriate medication, *VD* Vaud, *Top 5* Percentage of selected PIM class corresponding to the five most prevalent PIM classes at baseline, *DDD PIM/resident* Defined Daily Dose of PIM per resident as presented in the method section, No. *PIM class* number of PIM class selected

### Perspectives of maintenance

11/12 physicians responding to the follow-up survey, believed they had sustainably changed some of their prescribing practices because of the QC-DeMo session.

Furthermore, 8/12 physicians and 9/16 nurses would like to have a QC-DeMo session repeated at follow-up. Out of the 13 responding pharmacists, five did not plan to repeat a QC-DeMo session in the year following the session, while four pharmacists planned to repeat a session and four were unsure.

None of the active nursing homes renewed a dedicated QC-DeMo session but 11 nursing homes rediscussed the defined consensus one year after the initial QC-DeMo session during the annual review of the drug use. Two years after the intervention, 16 nursing homes reduced their PIMs compared to baseline. Differences in PIM reduction ranged from 0.03 to 0.67 DDD of PIM per average resident.

### Implementation strategies

#### Interprofessionality

After one year of implementation, physicians (*n* = 13) rated the degree of involvement and support of the nurses and pharmacist with a mean (± SD) score of 9.0 ± 1.0 out of 10. Nurses (*n* = 16) rated the degree of involvement and support with a mean score of 8.0 ± 1.5 for physicians and 8.4 ± 1.7 for pharmacists. Finally, pharmacists (*n* = 12) rated the degree of involvement and support with a mean score of 8.1 ± 1.8 for physicians and 8.4 ± 1.5 for nurses. Most of nurses and physicians also indicated that the interprofessionality was an essential part of the intervention 46/52 for nurses and 33/39 for physicians. Interprofessional collaboration was also explored by the perceived roles of each HCP at baseline. Most HCPs agreed that the role of physicians was major in establishing and applying the consensus, while nurses had a major role in follow-up measures. HCPs agreed that the pharmacists had a major role in co-establishing the consensus with physicians. The results are presented in Table 5.

**Table 5:** HCP’s role(s) in the deprescribing approach

	Deprescribing process								
	establishing consensus			application of the consensus			follow-up of deprescription measures		
HCPs role	Nurse’s perception n (%)	Physician’s perception n (%)	Pharmacist’s perception n (%)	Nurse’s perception n (%)	Physician’s perception n (%)	Pharmacist’s perception n (%)	Nurse’s perception n (%)	Physician’s perception n (%)	Pharmacist’s perception n (%)
**Nurses**	*n* = 51	*n* = 37	*n* = 19	*n* = 50	*n* = 36	*n* = 20	*n* = 49	*n* = 36	*n* = 20
Major	18 (35)	7 (19)	3 (16)	**26 (52)**	16 (44)	**11 (55)**	**36 (73)**	**20 (56)**	**12 (60)**
Important	**30 (59)**	**21 (57)**	**10 (53)**	23 (46)	**17 (47)**	5 (25)	12 (24)	15 (42)	8 (40)
Minor	3 (6)	8 (22)	6 (32)	1 (2)	3 (8)	2 (10)	1 (2)	1 (3)	0 (0)
No opinion	0 (0)	1 (3)	0 (0)	0 (0)	0 (0)	2 (10)	0 (0)	0 (0)	0 (0)
**Physicians**	*n* = 52	*n* = 37	*n* = 20	*n* = 49	*n* = 36	*n* = 20	*n* = 49	*n* = 36	*n* = 20
Major	**42 (81)**	**22 (59**)	**12 (60)**	**43 (88)**	**24 (67)**	**17 (85)**	**36 (73)**	16 (44)	7 (35)
Important	10 (19)	14 (38)	7 (35)	6 (12)	12 (33)	3 (15)	13 (27)	**19 (53)**	**11 (55)**
Minor	0 (0)	0 (0)	1 (5)	0 (0)	0 (0)	0 (0)	0 (0)	1 (3)	2 (10)
No opinion	0 (0)	1 (3)	0 (0)	0 (0)	0 (0)	0 (0)	0 (0)	0 (0)	0 (0)
**Pharmacists**	*n* = 51	*n* = 37	*n* = 20	*n* = 49	*n* = 35	*n* = 19	*n* = 50	*n* = 34	*n* = 20
Major	**33 (65)**	**20 (54)**	**12 (60)**	16 (33)	9 (26)	2 (11)	**22 (44)**	13 (38)	2 (10)
Important	15 (29)	15 (41)	7 (35)	15 (31)	**15 (43)**	7 (37)	16 (32)	**14 (41)**	**9 (45)**
Minor	3 (6)	2 (5)	1 (5)	**17 (35)**	8 (23)	**8 (42)**	12 (24)	4 (12)	**9 (45)**
No opinion	0 (0)	0 (0)	0 (0)	1(2)	3 (9)	2 (11)	0 (0)	3 (9)	0 (0)

*HCP* healthcare professional

According to physicians and nurses, the pharmacist's role in the deprescribing process was to identify eligible patients (Table 6). The common description of the nurses' role by pharmacists and physicians was immediate application of the consensus and daily monitoring of the residents (Table 6).

**Table 6:** HCP’s role in the deprescribing approach

Perception:	from physicians	from pharmacists	from nurses
Physicians Role description		• Deciding what to deprescribe • Implementation of deprescribing • Explaining the changes to residents and families	• Follow-up of patients • Discussions/reflections on treatment
Pharmacists Role description	• Identification of eligible patients • Coordination with physicians • Provision of scientific literature • Updating physician’s knowledge • Recommendation on the use of drugs, treatment recommendations • Participation in the medical visit to propose the deprescription to patients		• Identification of eligible patients • Reminding NH team of the consensus during meetings • Ensuring the implementation
Nurses Role description	• Daily follow-up, documentation, and feedback • Increasing physician’s awareness • Participation to the meetings • Immediate application of consensus	• Follow-up patients • Immediate implementation of consensus • Informing & encouraging the physicians • Defining tasks between nurses to implement and follow-up the consensus • Supporting the whole process	

*NH* nursing home

#### Training for pharmacists

The training was well perceived by pharmacists with a score of 8 ± 1 (*n* = 21) out of 10. After the training, most pharmacists (16/21) reported that they felt comfortable to lead a QC-DeMo workshop session and to support the establishment of a consensus on a PIM to deprescribe. While 12 pharmacists found that all the topics covered were useful for facilitating the QC-DeMo session, nine pharmacists noted that they had to complement the training with some information from the literature. Eleven pharmacists used the slides as provided, while 10 pharmacists made some modifications before presenting them.

#### Tailored strategies defined by nursing homes to implement the consensus

**Fig. 2:**
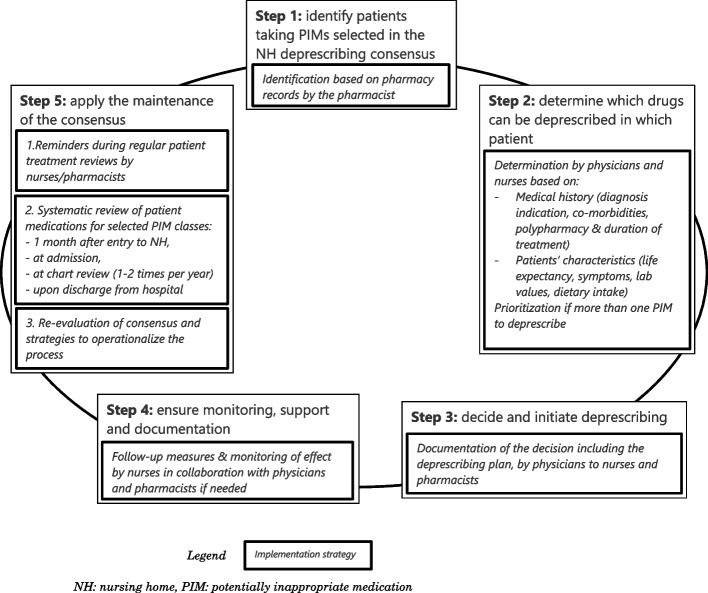
Tailored strategies defined by each nursing home team according to the steps of deprescription. Steps adapted from Reeve et al. [48]

The Fig. 2 summarizes the most relevant tailored strategies defined by each nursing home team during the QC-DeMo sessions based on the steps of the intervention.

## Discussion

This study describes the implementation results of a QC-DeMo intervention pragmatic RCT, including factors associated with successful implementation. We found that the QC-DeMo intervention was successfully implemented in terms of reach, fidelity, adoption, implementation, and implementation effectiveness. However, the intervention was implemented with low maintenance.

Adoption at the nursing home level was good for the first round, as it likely included the early adopters [49], but notably lower for the second round (35% vs 13%). One plausible explanation is the end of funding in the canton of Fribourg during 2018 for the remuneration of pharmacists, which is instrumental for the implementation and maintenance of the intervention. Interestingly, our results showed that, more than one-third of participating nursing homes, were already practicing, before the intervention, clinical activities to facilitate the deprescription of PIMs, such as medication reviews. This is reinforced by the fact that most physicians reported having already implemented deprescribing measures in their nursing home. These results suggests that most participating nursing homes were particularly motivated in these types of interventions before enrolling in the QC-Demo intervention.

Previous studies have demonstrated variation in implementation fidelity between nursing homes when delivering interventions [50–52]. In our study, even if fidelity was good, it did not guarantee an impact on PIM reduction, as the recommendation of choosing the most prevalent classes of PIMs were not clearly defined in the intervention. Thus, after analysis of the data showing a possible scattering of the intervention, we recommend to better define the intervention by choosing the three most prevalent PIMs classes used within a nursing home.

Most indicators showed good implementation with most HCPs finding easy to reach consensus and that they were satisfied with the QC-DeMo session and the overall approach. Their level of recommendation for others nursing homes to adopt a similar approach was also high. The level of importance to deprescribe in their nursing home was the highest for nurses, suggesting that they were the most convinced that this approach could be beneficial for their residents.

Given that, this intervention reached 824 residents in 26 nursing homes, the potential target that could be achieved if the intervention was expanded to all IPS nursing homes (*n* = 119) would be over 3770 potential residents. Thus, this intervention presents a good opportunity to target the patients at a lower cost than routine medication reviews.

According to the annual activities report of the IPS monitoring, none of the nursing homes of the canton of Vaud repeated a QC-DeMo session after the RCT, which means that it was not maintained over time. For future implementations, we suggest to add active facilitation, such as audit and feedback and sharing of practical experience through workshops [53] to harness the intervention into routine practice.

Indeed, facilitation is an implementation strategy that can influence the implementation and potentially the maintenance of an intervention. It can be defined as external support to help implement an intervention through addressing barriers such as changing peoples’ attitudes, habits, skills, ways of thinking and working. It is a process that depends upon the facilitator and them carrying out the role with appropriate skills, personal attributes, and knowledge [54, 55]. Organisational culture is also a known factor influencing the implementation and maintenance of interventions and should also be considered for future implementation [56].

Interestingly, physicians reported in the T12 questionnaire that they have sustainably changed their practice because of the QC-DeMo session. This suggests that the intervention may directly influence prescribing patterns in routine clinical practice. This seems to be confirmed in 16 out of 21 nursing homes of the Canton of Vaud, which had their PIM use reduced two years after the intervention. But we cannot detangle whether this result is directly related to the intervention or to another component such as the fact physicians would have reduced PIM use without the intervention.

Because of the low maintenance of the intervention observed in this study, we suggest that for future implementations, champions be better identified and engaged earlier in the implementation process [57], as one potential solution to better implement deprescribing interventions. Indeed, champions are defined as individuals (e.g. HCP, managers, or other staff members) who promote the implementation of a new intervention. The mechanism for how this implementation strategy works varies, for example it may be by them having a positive attitude towards it, by serving as team leader or by educating staff about the intervention [58, 59]. Thus, champions are known to effectively facilitate implementation and maintenance of a new intervention [57, 60]. Other solutions could also be considered, such as using the Normalization Process Theory [61] or tools such as NoMad [62].”

Given that we observed a large variation in PIM use reduction between nursing homes 2 years after the intervention, we suggest, as a future research perspective, to identify, whether there are champions in the nursing homes that reduced their PIM use the most. This would allow to explore e.g., the strategies they used to effectively reduce PIM use over the long term.

### Implementation strategies

The training course and material provided met expectations as it was rated highly by the pharmacists. We also observed that most pharmacists had not received training on deprescribing before the intervention. Interventions, such as QC-DeMo may be useful to train more pharmacists in the process of deprescribing medications. Indeed, they identified training and materials provided as factors facilitating implementation. Previous studies have indeed highlighted the effect of training to enhance implementation, although training alone is not enough to support full implementation [63–65]. In addition, we identified the need for reinforcing the training and the statistical support for pharmacists to measure the prevalence of PIMs in terms of DDD within their nursing home. Indeed, the prevalence of some PIM classes in nursing homes that selected those classes to be deprescribed, was particularly low (bisphosphonates and antidementia drugs). To improve training, it could be delivered as e-learning, which is more compatible with the community pharmacist activities. This allows for greater flexibility to follow the training and it is also a training format that facilitates the updating of the literature evidence.

Pre-existing interprofessional collaboration embedded in quality circles was also considered as a facilitator. The involvement and support of each HCP was rated as high. HCPs recognized a specific role for each HCP which actively contributes to the intervention. These results inform that interprofessional collaboration was already well established prior to the intervention.

In addition, most HCPs considered that each of them has at least one important role in the deprescribing process, except for pharmacist’s role. The most relevant tailored strategies to implement the deprescribing consensus were the identification of the patients by the pharmacist and a systematic review of patient medications. The tailored strategies of the consensus defined by the nursing home teams gave HCPs an opportunity to be more active in operationalising the intervention which is a recognized factor to improve implementation [42, 66]. Therefore, we encourage more active participation of HCPs in the design of clinical interventions to implement, while ensuring that general standards of care are respected.

### Strengths and limitations

This study has strengths. First, it assessed a multitude of exploratory implementation outcomes, which aimed to better describe how the intervention was implemented in practice and may better inform future implementation evaluations.

Secondly, the hybrid design of the pragmatic trial will save time for future implementations by providing insights into adoption, fidelity, reach, implementation and implementation effectiveness. Lastly, we had access to IPS monitoring data two years after the intervention and were able to compare these data with the data from the questionnaires. This allowed us to obtain additional quantitative information about maintenance through the rate of PIM and to compare it to data from the questionnaire on HCPs perception of maintenance.

In terms of limitations, this study was an observational study in a clinical research setting, embedded in a RCT. This context limited the ability to evaluate the implementation outcomes in all nursing homes of the IPS. The questionnaires we used were not validated. Missing data and the response rate were also a limitation to this study. Indeed, the response rate of the questionnaires at follow-up was low, particularly for nurses compared to HCPs who participated to the QC-DeMo session. We suggest, digital questionnaires to be used instead of paper ones to avoid the mailing process.

To design more robust studies, qualitative data obtained through interviews or focus groups rather than free text questionnaires would have increased the depth of responses and improved the ability to interpret the results.

Other limitations of the study are that each HCP’s understanding and description of their own role in the deprescribing process was not assessed and could not therefore be compared to the other HCPs to establish role congruence and dissonance for further implementation. Since each nursing home has developed its own tailored strategy through consensus, standardized deprescribing practices are difficult to generalize. Interprofessional collaboration was already established before starting QC-DeMo in participating nursing homes. Therefore, ideally for real-world implementation there would be a pre-existing interprofessional collaboration or alternatively as this may not exist in some nursing homes an implementation strategy to develop such collaboration would be needed. For our study, we used the FISpH framework to evaluate the implementation. It was chosen, after adapting it to our nursing home context, because it was usable in practice and was based on a widely used framework, the Consolidated for Implementation research Framework (CFIR). However, we could also have chosen a framework more specific to polypharmacy such as the one defined by Kherad et al. [67]. It has the advantage of being adaptable to the Swiss context and designed to help providers develop implementation strategies and implement corrective measures in clinical practice. The framework also considers the implementation of pre-graduate training to raise awareness of the problem of polypharmacy.

## Conclusions

Interprofessional quality circles on deprescribing in nursing homes were considered as well implemented in the context of our RCT. However, the choice of PIMs classes for deprescribing was not optimal. Pharmacists should be better trained and supported to identify the prevalence of PIMs in terms of DDDs within their nursing homes. A single implementation strategy is probably not enough to ensure sustainable implementation of the intervention in clinical practice. Additional implementation strategies should be provided within future RCTs and implementation efforts, such as active facilitation or training in a e-learning format.

### Supplementary Information

**Additional file 1: Annex 1. **Theme, items and corresponding type of scale of the questionnaires at baseline (T0) and follow-up (T12).

## Data Availability

The datasets generated during and analysed during this study, as well as the code used to analyse it, are available from the corresponding author upon reasonable request.

## Abbreviations

DDD: Defined Daily Dose
HCP: Healthcare professionals
IPS: Integrated Pharmacy Service
NH: Nursing Home
NORGEP-NH: Norwegian General Practice - Nursing Home criteria
OLD-NH: Opportunities and Limits to Deprescribing in Nursing Homes
PIM: Potentially Inappropriate Medication
PPIs: Proton Pump Inhibitors
QC: Quality Circle
QC-DeMo: Quality Circle Deprescribing Module
SD: Standard Deviation
