# Supplementary material for: Implementation of interprofessional quality circles on deprescribing in Swiss nursing homes: an observational study
Source: BMC Geriatr. 2023 Oct 3;23:620. doi: 10.1186/s12877-023-04335-w (PMC10548671; doi:10.1186/s12877-023-04335-w)
Supplement: Supplementary file 1 — Additional file 1: Annex 1. Theme, items and corresponding type of scale of the questionnaires at baseline (T0) and follow-up (T12). [file 12877_2023_4335_MOESM1_ESM.docx]

Annex 1: Theme, items and corresponding type of scale of the questionnaires at baseline (T0) and follow-up (T12)

| Questionnaire | Theme | Items | Type of scale |
| --- | --- | --- | --- |
| Baseline  T0 | Previous deprescribing experiences of HCPs | Number of years of collaboration within the NH | Numeric (free text) |
|  |  | Previous deprescribing training | Binary (yes/no) |
|  | Perception of implementing deprescribing measures within the NH | Degree of importance of implementing deprescribing measures within the nursing home | Score (0-10) |
|  |  | Deprescribing measures already implemented within the NH | Binary (yes/no) |
|  | Perception and satisfaction of HCPs concerning the QC-DeMo session they attended for nurses and physicians and led for pharmacists | Quality of the QC-Demo session | Score (0-10) |
|  |  | Comprehensiveness: quantity of information given during the QC-DeMo session | Useless, useful, abundant |
|  |  | Relevance: usefulness of the information given during the QC-DeMo session | Partially useless, partially useful, totally useful |
|  |  | Consensus: ease of establishment | Difficult, partially difficult, easy |
|  | Implementation strategy: training for pharmacists | Quality of the training for pharmacists | Score (0-10) |
|  | Implementation strategy: interprofessional collaboration | Importance of each HCPs role in the deprescribing process | Major, important, minor, no opinion |
|  |  | Importance of interprofessionality in this approach | unnecessary, useful, or essential |
| Follow-up  T12 | Evaluation of the process | Satisfaction | Score (0-10) |
|  |  | Barriers and facilitators to achieve the deprescribing consensus | Free text |
|  | Maintenance of the intervention | Proportion of physicians who believed they have sustainably changed some of their prescribing practices through the intervention | Binary (yes/no) |
|  |  | Proportion of physicians and nurses who would find a further QC-DeMo session useful | Binary (yes/no) |
|  |  | Proportion of pharmacists who intended to plan a second QC-DeMo session | Binary (yes/no) |
|  | Barriers and facilitators | Implementation of the consensus | Free text |
|  | Implementation strategy: interprofessional collaboration | Degree of involvement and support of each HCPs role | Score (0-10) |
|  |  | Description of the role of each HCP |  |
|  | Implementation strategy: operationalisation process | Description of the operationalisation process after a year | Free text |
|  |  | Effectiveness of the operationalisation process reported by HCPs | Score (0-10) |

HCP= health care professionals, NH=nursing home, QC-DeMo=Quality Circle Deprescribing Module, T0=questionnaire at baseline, T12: questionnaire at follow-up
